# Post-feeding Larval Dispersal Patterns of the Necrophagous Blowfly Lucilia sericata (Diptera: Calliphoridae) Associated to Swine Carcasses

**DOI:** 10.1093/jme/tjag085

**Published:** 2026-07-10

**Authors:** Nuple-Juárez Eduardo, Gabriela Castaño-Meneses, Johnattan Hernández Cumplido, Zenón Cano-Santana

**Affiliations:** Posgrado en Ciencias Biológicas, Unidad de Posgrado, Edificio A, 1° Piso, Circuito de Posgrados, Universidad Nacional Autónoma de México, Ciudad Universitaria, Ciudad de México, Mexico; Laboratorio de Interacciones y Procesos Ecológicos, Departamento de Ecología y Recursos Naturales, Facultad de Ciencias, Universidad Nacional Autónoma de México, Ciudad Universitaria, Ciudad de México, Mexico; Laboratorio de Ecología de Artrópodos en Ambientes Extremos, Unidad Multidisciplinaria de Docencia e Investigación (UMDI), Facultad de Ciencias, Universidad Nacional Autónoma de México, Juriquilla, Querétaro, Mexico; Laboratorio de Interacciones y Procesos Ecológicos, Departamento de Ecología y Recursos Naturales, Facultad de Ciencias, Universidad Nacional Autónoma de México, Ciudad Universitaria, Ciudad de México, Mexico; Laboratorio de Interacciones y Procesos Ecológicos, Departamento de Ecología y Recursos Naturales, Facultad de Ciencias, Universidad Nacional Autónoma de México, Ciudad Universitaria, Ciudad de México, Mexico

**Keywords:** directional behavior, forensic entomology, *Lucilia sericata*, post-feeding dispersal

## Abstract

Accurate minimum post-mortem interval estimation in forensic entomology relies on understanding necrophagous dipteran dispersal behavior. Post-feeding larval dispersal represents a critical phase impacting evidence recovery in forensic research. Currently, most studies suggest uniform radial dispersal patterns. Our study quantified spatial and temporal patterns of post-feeding larval dispersal in necrophagous Diptera under field conditions, examining how temperature, humidity, soil and season influence dispersal distance and directionality from a dead body. Twelve swine carcasses were deployed across wet and dry seasons in the Pedregal de San Ángel Ecological Reserve, Mexico City. Pitfall traps arranged in 9 concentric rings (1 to 7.4 m diameter) with 36 angular sectors monitored larval dispersal patterns. Among 2,545 individuals from 10 morphospecies were recorded, *Lucilia sericata* (Meigen) was the most dominant species (87.04%). Circular statistics rejected uniform distribution, revealing significant northwest directional preference (320.74°). Spatial distribution exhibited bimodal patterns with 50% of larvae within 130 cm and 95% within 290 cm. Dry season showed higher larvae concentration toward northwest compared to wet season. Predation by *Solenopsis* sp. Westwood ants during dry season forced larval aggregation in situ. Post-feeding larval dispersal exhibits consistent directional bias, complementing current uniform radial models. Our findings optimize entomological evidence collection by establishing search priorities: primary exhaustive searches within 3 m capture 95% of dispersing larvae, directional searches toward northwest up to 5 m increase recovery during wet seasons, and concentrated searches within 0.5 m maximize recovery during dry conditions when predation restricts dispersal. These quantified metrics provide operational guidelines for evidence recovery in Neotropical forensic investigations, improving search efficiency while reducing investigation time.

## Graphical Abstract

**Graphical Abstract:**
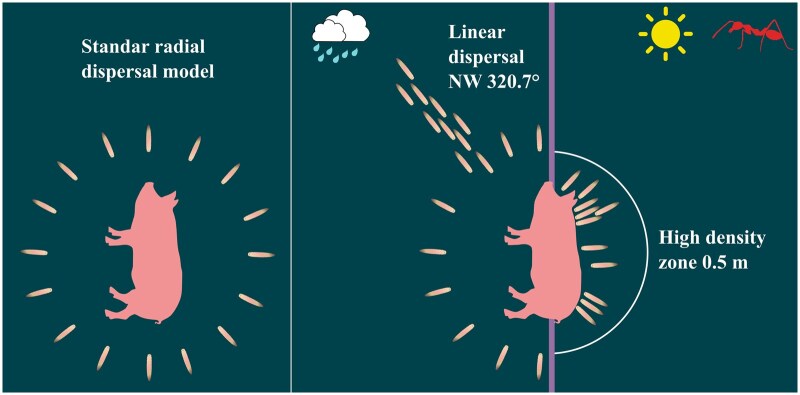

## Introduction

Necrophagous dipterans, particularly species within the Calliphoridae family, constitute important tools in forensic entomology due to their predictable successional colonization and developmental timelines on decomposing remains (Byrd and Tomberlin 2020, Singh et al. 2025). These insects are typically among the first colonizers during invertebrate succession on decomposing animal remains, including humans, with oviposition occurring often within the first 6 h of exposure (Matuszewski et al. 2011, Owings et al. 2025). From an investigative perspective, the successful recovery of entomological evidence relies heavily on understanding dispersal behavior and pupariation site selection (Arnott and Turner 2008, Sharma et al. 2015). Locating these dispersing individuals is a critical prerequisite for subsequent forensic analyses, such as accurate minimum post-mortem interval (minPMI) estimations, which ultimately rely on the recovery of the most advanced developmental stages (Turpin et al. 2014, Mohr and Tomberlin 2015).

After completing their feeding phase on carrion sources, third-instar dipteran larvae enter a stage termed post-feeding dispersal in which the search for an ideal pupariation site and avoidance of intraspecific competition drive them to abandon feeding areas in search of microenvironments that minimize their exposure to predators and environmental stresses (Greenberg 1990). In this context, pupariation site selection is influenced by a myriad of signals ranging from intrinsic physiological triggers to environmental cues (Gomes et al. 2006, Mactaggart et al. 2025). Among these, temperature gradients are particularly important. Because their development is highly temperature-dependent, larvae rely on behavioral thermoregulation to actively navigate thermal gradients, avoiding species-specific lethal extremes (Grassberger and Reiter 2001, Aubernon et al. 2019) and selecting areas where environmental conditions are conducive to rapid puparium sclerotization and metamorphosis (Komo et al. 2020). Dipteran larvae generally prefer temperatures between 20 and 25°C (Lavezzo et al. 2023) and substrates that offer protection or are porous (Robinson et al. 2018), which must be sufficiently dry to prevent fungal or bacterial infestation, yet sufficiently moist to avoid desiccation of the delicate puparium (Komo et al. 2020).

Larval movement orientation during post-feeding dispersal phase is typically characterized by radial outward movement from the carrion source (Gomes et al. 2006, Turpin et al. 2014). Several studies have documented larvae consistently disperse radially from carcasses in all directions, a typical pattern that likely minimizes intraspecific competition and increases the probability of locating an optimal pupariation site (Gomes et al. 2006, Komo et al. 2020). However, mass dispersal has also been documented where larvae on the carcasses move simultaneously in the same direction as 1 or 2 collective aggregates, but with contrasting orientations across different calliphorid species. For example, eastward movement in *Lucilia coeruleiviridis* Macquart (Goddard et al. 2020), southeast dispersal in *Chrysomya albiceps* (Wiedemann) and *Ch. chloropyga* (Wiedemann) (Adetimehin et al. 2024), southeast to southwest patterns for assemblages including *Cochliomyia macellaria* (F.) (Tessmer and Meek 1996), while other studies document north to northeast movement for *Phormia regina* (Meigen) (Lewis and Benbow 2011). This specific directional variation among Calliphoridae demonstrates that no consensus exists yet regarding movement orientation patterns, and comprehensive knowledge about dispersal directions in natural environments remains still limited.

While spatial orientation dictates the vector of larval movement, the total dispersal distance is heavily constrained by local abiotic factors, primarily soil composition, moisture, and temperature. Alongside direction, distance acts as a highly plastic, adaptive response to the physical properties of the substrate (Gomes et al. 2006). Soil texture and porosity serve as primary physical determinants (Bambaradeniya et al. 2023, Hans and VanLaerhoven 2024); compact or hard substrates prevent immediate burrowing, forcing larvae to undergo prolonged dispersal phases that consume significant energy reserves, ultimately resulting in smaller puparia and reduced adult body sizes (Mai and Amendt 2012, Robinson et al. 2018). Conversely, loose and porous soils facilitate rapid pupariation, drastically restricting horizontal travel (Vogt and Woodburn 1982). Furthermore, precipitation and soil moisture significantly alter these dynamics. High moisture levels and soil saturation displace oxygen, preventing pupariation directly beneath the carrion and forcing larvae to migrate further in search of adequately drained environments to avoid asphyxiation (Kökdener and Yurtgan 2022). In contrast, encountering dry, optimal substrates can rapidly terminate the post-feeding wandering phase (Thümmel et al. 2024), provided larvae can successfully evade desiccation. Temperature extremes additionally modulate this traveled distance; driven by behavioral thermoregulation, larvae facing lethal surface heat must extend their horizontal migration to shaded refuges or increase their vertical burrowing depth to ensure survival (Marchenko 2001, Tarone et al. 2011).

Despite these studies, uncertainties persist regarding how heterogeneity in abiotic variables, as well as interspecific interactions, influence larval dispersal patterns. Due to the scarcity of studies analyzing dispersal processes under field conditions, this study aims to examine dispersal patterns of necrophagous dipteran larvae from swine carcasses based on orientation and distance from the cadaver in a Neotropical environment within the Valley of Mexico. This region exhibits a marked seasonal precipitation regime: a dry season (November to April) characterized by water stress, and a wet season (May to October) that concentrates over 70% of the annual precipitation (Vega et al. 2023, Muñoz-Sanchez et al. 2024). This stark seasonal contrast profoundly modulates soil arthropod dynamics and substrate conditions (Razo-González et al. 2014), providing an ideal scenario to test environmental constraints on larval movement. Understanding these constraints holds significant practical importance for forensic investigations, as optimizing the recovery of dispersed puparia is essential for accurate minimum post-mortem interval (minPMI) estimations (Byrd and Tomberlin 2020). We hypothesize that dispersal will occur radially and that temperature and soil will modify the distance at which larvae pupate from the cadaver. Furthermore, we predict that porous soil conditions and wet seasons will promote shorter dispersal distances, maintaining larvae closer to the cadaver.

## Materials and Methods

### Study Area

**Fig. 1.**
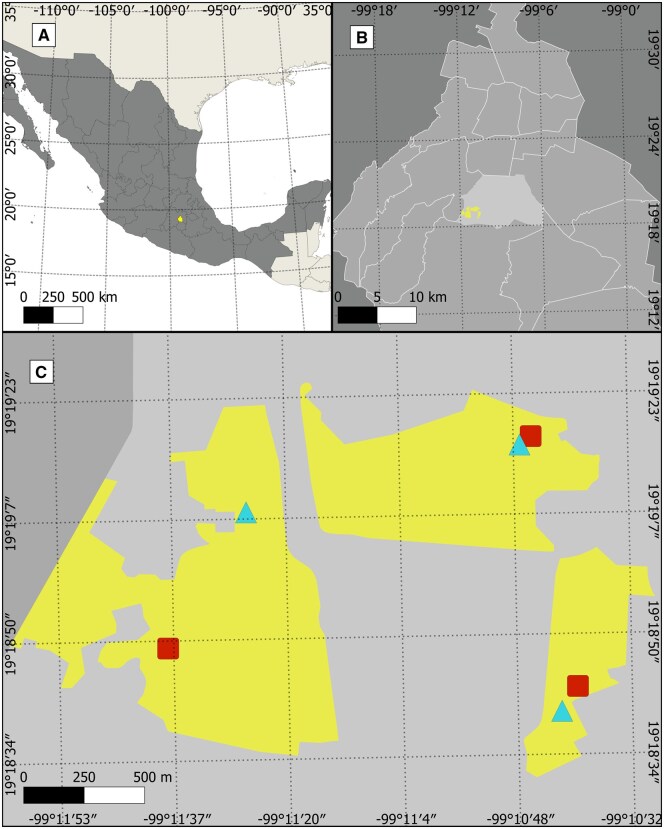
Geographic location of the study sites within the Pedregal de San Ángel Ecological Reserve (PSAER), Mexico City. (A) Location of the study area within Mexico. (B) Location within Mexico City. (C) Specific sampling sites within the yellow polygons of the reserve, with light blue triangles indicating wet season collection sites and dark red squares representing dry season collection sites.

This study was conducted in the Pedregal de San Ángel Ecological Reserve (PSAER), located within the main campus of the Universidad Nacional Autónoma de México (UNAM) at southern Mexico City (19°18'-19°20'N, 99°10'-99°12'W) at elevations between 2,270 and 2,380 m above sea level (Castillo-Argüero et al. 2007). PSAER encompasses 264 ha as an insular ecosystem surrounded by urbanization, with volcanic soils derived from the eruption of Xitle volcano, characterized by rocky terrain and heterogeneity due to historical landfill processes over the original basaltic rock, resulting in a complex matrix of volcanic soils, basaltic rock fragments, and dispersed anthropogenic materials (Zambrano and Cano-Santana 2016, UNAM 2025). Three flat experimental sites with sparse vegetation were selected to facilitate carcass placement and larval visualization during post-feeding dispersal. These sites were named according to their location within the PSAER’s core area: East, West, and Southeast sites (Fig. 1).

### Experimental Setup and Larval Dispersal Monitoring

Twelve swine carcasses (*Sus scrofa* L.) weighing 12.6 to 14.1 kg were deployed during 2 seasonal periods: wet season (August to October 2022 and 2023, *n* = 6) and dry season (March to June 2023 and 2024, *n* = 6). Animals were euthanized by exsanguination approved by the Institutional Committee for the Care and Use of Laboratory Animals (CICUAL, FMVZ-UNAM) in compliance with NOM-033-SAG/ZOO-2014 (SAGARPA 2015). Furthermore, field interventions at PSAER were approved by the Bioethics Subcommittee of the Facultad de Ciencias, UNAM (Protocol: PI_01_04_2024_Cano). Immediately after euthanization, carcasses were placed in individual plastic bags and transported in refrigerated containers to minimize decomposition during transport. Following an approximate transport period of 4 h, field deployment occurred between 12:00 and 13:00 h (local time) at each study site to standardize colonization timing. To structure the temporal and spatial replicates, 3 carcasses were deployed simultaneously during each seasonal trial, placing exactly one carcass within each of the 3 established sites (East, West, and Southeast). This design provided 2 temporal replicates per season across different years (eg wet season 2022 and wet season 2023), resulting in a total of 6 carcasses per season and 12 carcasses overall. Carcass orientation was standardized with the snout directed north and the ventral region oriented west to minimize directional variability among replicates. Each carcass was covered with a wire mesh cage (1.25 cm aperture) to prevent disturbance by vertebrate scavengers while allowing unrestricted access to colonizing arthropods.

**Fig. 2.**
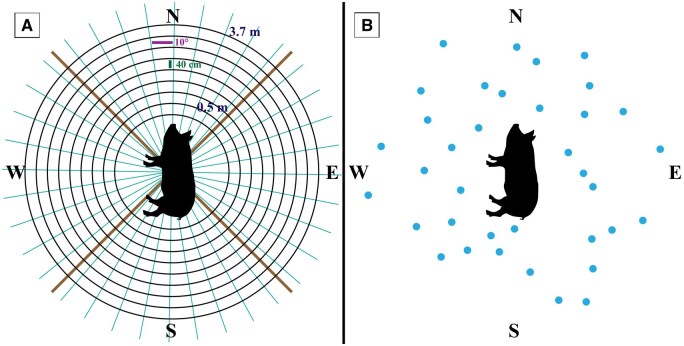
Schematic representation of the sampling designs employed around the carcass. (A) Systematic sampling grid illustrating concentric circles spaced at 0.5 m intervals up to a 3.7 m radius, divided by radial lines every 10°. The green bar indicates a 40 cm scale reference, and the purple bar shows the 10° angular separation. Thick brown lines indicate the main transect axes. (B) Random sampling design showing the distribution of collection points (blue dots) around the central pig silhouette. Cardinal directions (N, S, E, W) are indicated in both panels.

Around each carcass, a pitfall trap web system was established, using 500 ml capacity containers. The system was organized into 9 concentric rings with progressive diameters from 1.0 to 7.4 m and uniform 40 cm separation between consecutive rings (Fig. 2). To enable precise monitoring of dispersal directions toward pupariation sites, each ring was virtually segmented into 36 sectors of 10°. A total 36 pitfall traps were deployed per carcass. Four traps were placed within each of the 9 concentric rings. To ensure balanced spatial coverage, these 4 traps were distributed with exactly one trap assigned to each cardinal orientation (North, South, East, and West) per ring, resulting in 9 traps per cardinal direction overall. Within its assigned cardinal quadrant, the exact placement of each trap was randomized among the available 10° sectors. This staggered, nonlinear spatial configuration was specifically designed to prevent the formation of artificial radial barriers that could prematurely intercept long-distance dispersing larvae. Crucially, this randomized layout was uniquely generated for each of the 3 experimental sites, ensuring that trap placement was ecologically tailored to each location; however, the same site-specific configuration was consistently replicated across both seasonal trials (wet and dry) at that site, thereby allowing rigorous seasonal comparisons within each location.

Traps consisted of plastic containers (10 cm diameter × 7 cm depth) buried at ground level, containing 50% ethanol solution and were replaced weekly for 6 wk, representing the standard period of post-feeding larval dispersal for necrophagous arthropods. Given that all spatial replicates were standardized to this 6-wk period, the combined sampling effort yielded a cumulative total of 435,456 trap-hours (432 pitfall traps × 168 h per week × 6 wk). Rather than representing linear time, this aggregate metric establishes a standardized baseline for evaluating larval capture rates. All specimens were collected and preserved in 70% ethanol and transported to the laboratory for taxonomic identification to species level using taxonomic keys (Wells et al. 1999, Florez and Wolff 2009, Thyssen 2010, Velázquez et al. 2010, Szpila et al. 2015, Díaz-Aranda et al. 2018). Larval abundance data were recorded per individual trap, including zero abundance observations to reflect the complete spatial structuration of the dispersal process. Additionally, daily direct qualitative behavioral observations of larvae were conducted during 10-min intervals per carcass. Data from these observations were used to document mass dispersal events and biotic factors such as predation, providing the necessary ecological context to explain the spatial restriction and aggregation patterns detected in the quantitative trap data. Observations involved systematic visual inspection of all carcass regions (head, thorax, abdomen, and limbs) without disturbing the carcass. Observation timing followed across 3 daily periods (07 to 09, 09 to 11, and 14 to 16 h), with site visitation order rotating systematically (East, West, and Southeast). Observation frequency was daily during the first 3 wk, reduced to 3 times per week during week 4, and continued every 3 d until skeletal stage (weeks 5 to 6). Total observation effort was 168 h across all carcasses and sampling periods.

### Substrate Characterization

To analyze the relationship between apparent soil density and larval dispersal variables, soil samples were obtained for bulk density characterization at each of the 6 experimental sites. Sampling was conducted retrospectively in August 2025. In undisturbed volcanic soils with no history of mechanical tillage, bulk density is primarily governed by parent material composition, and interannual variation has been reported in the range of 3% to 7% in comparable monitoring contexts (Berhe 2019, Širáň and Makovníková 2021). These measurements are therefore considered a reasonable approximation of baseline substrate structural conditions. A cylindrical PVC corer (2.5 cm height × 3.5 cm diameter) was used to extract samples of known volume (19.1 cm³) from substrate surrounding each carcass, avoiding areas with exposed basaltic rock or anthropogenic materials. Following the protocol established by Siebe et al. (2016), samples were oven-dried at >100°C for 24 h to eliminate residual moisture, then weighed on an analytical balance to determine dry mass. Soil bulk density was calculated using the formula: Bulk density (g/cm³) = Dry soil mass (g)/Sample volume (cm³). Two independent samples per site were obtained for local spatial variability assessment.

### Microclimatic Conditions

Microclimatic conditions were continuously recorded using dataloggers HOBO U23 Pro v2 External Temperature Data Logger installed within each protective wire cage. Sensors recorded ambient temperature (°C) and relative humidity (%) at 5-min intervals. To accurately reflect the environmental conditions experienced by the insects, daily minimum, maximum, and mean values were extracted and analyzed exclusively for periods encompassing active larval dispersal, extended through a standardized post-activity buffer to the end of the sampling period for each carcass. For statistical analyses, daily data were averaged weekly within these active sampling periods.

### Data Analysis

Following taxonomic identification and abundance table construction by species, a rank-abundance plot was constructed to evaluate total larval community structure. Based on relative abundance and established forensic relevance in the literature, *Lucilia sericata* was selected as the focal species for detailed dispersal pattern analyses (87.04% of the total larval abundance). Data were temporally aggregated to prevent pseudoreplication, maintaining carcass as the independent replicate unit. Angular data were organized into 36 sectors of 10° and weighted by larval abundance for subsequent circular analyses. Statistical analysis was structured hierarchically following 4 complementary research questions to evaluate dispersal patterns progressing from general to specific aspects:

#### Directional Analysis

Circular statistics were applied to evaluate directional preference *versus* uniform distribution. Angular data were weighted by larval abundance to calculate the mean resultant vector and its length (*r*). The weighted Rayleigh test was applied (*H_0_*: uniform distribution vs *H_1_*: preferred directionality), calculated as *Z = nr*^2^, where *n* represents total abundance and *r* the resultant vector length. Circular descriptive statistics were calculated including directional mean, concentration parameter (κ), and circular standard deviation.

#### Radial Distribution Analysis

Area-corrected chi-square test was employed to address inherent geometric heterogeneity in the circular design. Expected frequencies were adjusted by actual sampling area in each concentric ring, applying correction for effective trap radius (5.5 cm). Dispersal metrics were calculated including weighted mean distance, *D*_50_ (distance containing 50% of larvae), and *D*_95_ (95% of larvae). Because the four 10-cm-diameter traps per ring collectively occupy a negligible fraction of the total circumference at any given radius, inner rings are unlikely to act as an artificial interception barrier for larvae traveling to outer distances. This design feature, combined with the staggered randomization within quadrants, minimizes spatial bias in dispersal distance estimates.

#### Spatial Variation Analysis

Kruskal–Wallis test was used to compare abundances among the 3 sites (East, West, and Southeast), employing carcass as independent replicate unit. Additionally, soil bulk density was compared among sites using a Kruskal–Wallis test to assess substrate homogeneity, as this nonparametric method is appropriate for the limited sample size per site (*n* = 2) and the high environmental heterogeneity of the area. Independent resultant vectors were calculated by site to evaluate spatial consistency of directional patterns.

#### Temporal Variation Analysis

Mann–Whitney *U* test was applied to compare abundances between wet and dry seasons. Directional patterns were analyzed by season through independent resultant vectors.

All analyses were executed in R v4.3.0 (R Core Team 2023) within RStudio (RStudio Team 2023) using “circular” (Agostinelli and Lund 2023) and “CircStats” (Agostinelli and Lund 2022) packages for circular statistics, “tidyverse” (Wickham et al. 2019) for data manipulation, and “ggplot2” (Wickham 2016) for visualizations. A significance level of *P* = 0.05 was employed for all hypothesis tests.

## Results

### Necrophagous Dipteran Community Structure

During sampling across 4 seasons over 6 wk of cadaver decomposition, a total of 2,545 specimens were captured, with a seasonal total abundance of 2,297 in the rainy season and 248 in the dry season. The mean capture rate was 848.3 specimens per site. These individuals represented 10 taxa distributed across 6 families within 3 taxonomic orders (Table 1). Taxonomic structure revealed Diptera dominance (94.1% of total abundance) represented by 7 species, followed by Coleoptera with one taxon belonging to Staphylinidae (Silphinae), while lepidopteran larvae were considered as a single group due to their incidental occurrence.

**Table 1.** Abundance and taxonomic richness of necrophagous arthropods captured during post-feeding dispersal from swine carcasses, PSAER (2022 to 2024)

Order	Family	Taxa	*N*	Relative abundance (%)
**Diptera**	Calliphoridae	*Lucilia sericata*	2,215	87.04
**Coleoptera**	Staphylinidae	*Necrodes littoralis*	134	5.26
**Diptera**	Piophilidae	*Piophila casei*	119	4.68
**Diptera**	Calliphoridae	*Chrysomya rufifacies*	29	1.14
**Diptera**	Calliphoridae	*Chrysomya megacephala*	24	0.94
**Lepidoptera**		Lepidoptera	15	0.59
**Diptera**	Fanniidae	*Fannia scalaris*	4	0.16
**Diptera**	Sarcophagidae	Sarcophagidae	3	0.12
**Diptera**	Calliphoridae	*Lucilia* sp.	1	0.04
**Diptera**	Phoridae	Phoridae	1	0.04

*L. sericata* (Calliphoridae) constituted the dominant taxon with 2,215 individuals (87.04% relative abundance), establishing a highly dominant community structure toward this species. Secondary taxa, *Necrodes littoralis* (L.) (Staphylinidae, 5.26%) and *Piophila casei* (L.) (Piophilidae, 4.68%), jointly represented 9.94% of the community. Based on its numerical dominance and established forensic relevance, *L. sericata* was selected as the focal species for detailed post-feeding dispersal pattern analyses.

### Environmental Conditions Characterization

#### Seasonal Climatic Conditions

Microclimatic conditions showed marked differences between experimental seasons. The wet season (*n* = 10 active sampling weeks pooled across 2022 and 2023) exhibited mean temperatures of 18.1 ± 1.4 °C. Daily maximum temperature ranges of 28.7 to 37.6°C, while daily minimum temperatures tanged from 2.9 to 10.8 °C. Mean relative humidity was 74.9 ± 7.2%, with maximum values consistently near saturation (99.1 to 100.0%) and minimums of 17.5 to 38.0%.

Dry season (*n* = 11 active sampling weeks pooled across 2023 and 2024) exhibited mean temperatures of 18.5 ± 2.4 °C, presenting more extreme thermal fluctuations. Daily maximum temperature ranges from 23.6 to 45.8 °C, while daily minimum reaching −6.5 °C to 6.7 °C. Mean relative humidity was significantly lower (50.1 ± 11.8%), with maximum values of 53.4 to 100.0% and minimums of 4.8 to 20.8%, indicating conditions of greater water stress during this season.

#### Substrate Properties

Soil bulk density showed considerable variation among sites and seasons (range: 0.49 to 2.21 g/cm³). No statistically significant differences were detected among sites in either the rainy season (*H* = 4.57, df = 2, *P* = 0.1) or the dry season (*H* = 4.57, df = 2, *P* = 0.1). Mean values (± SE) by site and season were East wet (0.76 ± 0.02 g/cm³), East dry (0.97 ± 0.03 g/cm³), West wet (0.89 ± 0.1 g/cm³), West dry (0.832 ± 0.02 g/cm³), Southwest wet (1.92 ± 0.3 g/cm³), and Southwest dry (0.49 ± 0.01 g/cm³). The high variability, particularly in the southwest site during the rainy season, reflects the characteristic heterogeneity of the volcanic substrate within PSAER.

### Directional Dispersal Patterns in *L. sericata*

**Fig. 3.**
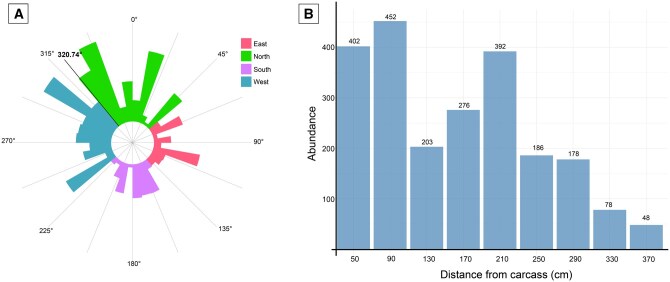
Spatial patterns of *Lucilia sericata* post-feeding dispersal in a Neotropical volcanic substrate. (A) Circular histogram displaying the directional frequency of larval dispersal, highlighting a significant northwest orientation with an angular mean of 320.74° (black line). Colors denote the cardinal quadrants: North (green), East (pink), South (purple), and West (blue). (B) Histogram of larval abundance by distance from the carcass, demonstrating a bimodal distribution with peaks at 90 and 210 cm.

Circular statistical analysis applied to 2,215 *L. sericata* larvae rejected the uniform distribution hypothesis (weighted Rayleigh test: *Z* = 147.29, *P* < 0.05), revealing a significant directional dispersal pattern. The mean dispersal direction was calculated at 320.7° ± 94.3° (circular standard deviation), indicating a northwest orientation. The resultant vector length *r* = 0.258 indicate moderate directional concentration (Fig. 3, left). Circular descriptive statistics confirmed the non-random nature of dispersal: concentration parameter κ = 0.63. In circular statistics, κ measures the degree of clustering around the mean direction; a value of zero represents a uniform, random distribution, while higher values indicate stronger directional aggregation. These values demonstrate that post-feeding *L. sericata* larvae exhibit statistically robust preference toward the northwest sector.

Distance distribution analysis, corrected for available area in each concentric ring, revealed a significantly heterogeneous pattern (χ^2^ = 3,709.56, df = 8, *P* < 0.001). The distribution exhibited bimodal characteristics with abundance peaks at 90 cm (452 larvae, 20.4%) and 210 cm (392 larvae, 17.7%), followed by exponential decay toward greater distances (Fig. 3, right). Quantitative dispersal metrics established operational limits for forensic searches: weighted mean distance = 161.6 cm, dispersal median (*D_50_*) = 130 cm, and 95th percentile (*D_95_*) = 290 cm. These results indicate that 50% of larvae pupate within 1.3 m of the cadaver, while 95% concentrate within 2.9 m, establishing effective limits for entomological evidence recovery protocols.

### Spatial and Temporal Differences

Spatial variation analysis detected no significant differences in larval abundance among the 3 study sites (*H* = 1.703, df = 2, *P* = 0.427), indicating homogeneity in colonization processes at landscape scale within PSAER. Median abundances by site were: East (702 larvae), Southeast (1,141 larvae), and West (374 larvae). Directional analysis revealed spatial consistency in northwest preference, with mean directions by zone: East 338° (*r* = 0.292), West 303° (*r* = 0.352), and Southeast 317° (*r* = 0.221). Despite variations in directional concentration magnitude (range *r* = 0.221 to 0.352), preferential orientation remained spatially robust, suggesting influence of consistent environmental factors at study site scale.

Temporal comparative analysis revealed no significant differences in total *L. sericata* abundance between seasons (*W* = 12.5, *P* = 0.437). The wet season recorded a median of 200 larvae per carcass (interquartile range: 31 to 534, *n* = 6 carcasses), while the dry season presented 53 larvae per carcass (interquartile range: 28 to 62, *n* = 6 carcasses). Despite this nearly 4-fold difference between medians, the variation lacked statistical significance due to the extreme intra-seasonal data dispersion (evidenced by the wide interquartile range of the wet season) and the limited number of spatial replicates. Regarding the temporal dynamics of *L. sericata* dispersal, significant variation was noted between seasons. During the wet season, post-feeding dispersal began promptly, with activity recorded during the first 3 wk of the experimental period in 2022, and weeks 1, 2, and 4 in 2023. In contrast, the dry season exhibited a delayed colonization pattern; no dispersing larvae were captured during the first week of the experimental period in either 2023 or 2024. Active dispersal in the dry season was primarily concentrated from week 2 onwards, extending up to week 6 in 2023. Furthermore, marked differences emerged in seasonal directional patterns. The wet season showed dispersal toward 316° ± 95.6° (*r* = 0.248, *n* = 2,088 larvae), while the dry season oriented toward 357° ± 64.2° (*r* = 0.534, *n* = 127 larvae). The dry season exhibited notably superior directional concentration (*r* = 0.534 vs 0.248), suggesting more selective responses during restrictive environmental conditions.

### Season-Specific Behavioral Observations

#### Wet Season

Direct observations documented mass dispersal events of *Chrysomya rufifacies* (Macquart) between days 10 to 13 post-exposure, typically occurring between 09 and 11 h. These aggregations contained approximately 50 individuals organized in formations of 30 cm length × 12 cm width, moving directionally toward the northwest in synchronized manner. Also, during this season, *N. littoralis* larvae were present, always at distances less than 90 cm, with few cases at 170 cm (19 individuals), showing greater concentration toward the eastern zone. *Chrysomya megacephala* (F.) larvae were also found only during wet season and while few and concentrated at 50 cm, some reached various points up to 2.5 m (3 individuals) with orientation less toward the western orientation with only one individual. *Fannia scalaris* (F.) was found only during wet season at a maximum distance of 90 cm.

#### Dry Season

An altered dispersal pattern was observed, characterized by intensified larval aggregation under severe water stress conditions (mean humidity: 50.1%, minimums to 4.8%). Observations revealed significant predatory activity by *Solenopsis* sp. ants with activity radius of 1.7 m around each carcass, effectively limiting larval dispersal. Additionally, consistent avian activity was recorded around carcasses. As consequence of this predatory pressure, massive *in situ* pupariation was documented with >500 pupae per carcass distributed in aggregated radial patterns around specific body regions (head and abdomen). Pupal aggregations varied from 5 to 60 individuals in smaller groups and 200 to 300 in main aggregations, contrasting markedly with the dispersive pattern observed during wet season. Similarly, *P. casei* larvae were found only during these seasons after week 3, at 50 cm distance with some up to 210 cm (12 individuals), while their orientation also appears homogeneous but scarce toward the northern zone.

## Discussion

### Dispersal Pattern Synthesis

Understanding the post-feeding dispersal behavior of necrophagous Diptera is essential for accurate forensic entomology applications, as the spatial distribution of migrating larvae directly impacts the recovery of the oldest specimens used for minimum post-mortem interval estimations (Gomes et al. 2006). In this context, our study demonstrates that *L. sericata* post-feeding dispersal is characterized by a consistent northwest directionality (320°), although its intensity varies seasonally. Furthermore, we observed spatially limited dispersal—with 95% of pupariation occurring within 2.9 m of the cadaver. While pitfall arrays inherently carry a risk of prematurely intercepting some individuals (potentially underestimating absolute biological limits), our sparse web design and area-corrected statistical analysis minimize this bias, making this 2.9 m threshold a highly robust, conservative operational limit for entomological evidence recovery. This spatially limited dispersal occurred alongside spatial homogeneity among sites and temporal plasticity in response to seasonal predatory pressure. Thus, these quantified patterns provide valuable empirical baseline data, adding critical information about larval movement and post-feeding behavior in Neotropical environments.

### Taxonomic Limitations and Regional Diversity

Although the vast majority of dispersing larvae were successfully identified to species level, a small fraction of specimens—including one *Lucilia* sp. and 3 Sarcophagidae—remained unresolved, exposing a systemic limitation: identification keys available for forensically relevant Diptera were developed primarily for Palearctic and Nearctic fauna and are inadequate for Neotropical diversity, posing a risk of misclassification when applied to Mexican assemblages. This gap is particularly concerning for taxonomically complex groups such as Sarcophagidae. A recent study demonstrate that dispersal behavior is not universal but highly species-specific; within the same genus, *Peckia chrysostoma* (Wiedemann, 1830) exhibits strongly oriented, gregarious dispersal, whereas *Peckia lambens* (Wiedemann, 1830) moves randomly and multidirectionally (Ferreira et al. 2026). Consequently, the inability to identify Neotropical specimens to the species level directly compromises the ability to predict their spatial distribution, and likely underestimates cryptic diversity in the non-dispersing carcass community. The faunal composition documented here—including *Ch. megacephala*, *F. scalaris*, *P. casei*, and a first dispersal report of *N. littoralis*— diverges markedly from North American assemblages dominated by *Co. macellaria* or *P. regina* (ie Tessmer and Meek 1996), reinforcing the need for regionally specific taxonomic resources. Efforts exist in Latin America, such as the larval key for Colombian Calliphoridae developed by Florez and Wolff (2009) or an interactive key for Neotropical larval blow flies by Prado et al. (2023), but equivalent work for Mexico remains needed to ensure the reliability of forensic entomology applications in the region.

### Biogeographic Patterns and Directional Convergence

The northwest directional pattern documented for *L. sericata* represents one of the first quantitative field assessments of post-feeding larval dispersal in a Neotropical ecosystem. Current understanding exhibits marked Nearctic bias, with extensive temperate North American studies revealing environment-specific patterns rather than general behaviors. For example, in Louisiana wetlands (United States), Tessmer and Meek (1996) documented dispersal directions range from southeast to southwest for *L. sericata*, *P. regina*, and *Co. macellaria* regardless of species dominance, while Lewis and Benbow (2011) observed *P. regina* moving from 2 to 26 m north to northeast through Michigan deciduous forests during mass dispersal events triggered by soil saturation. Heinrich (2013) reported sunrise-oriented northeast movement for *P. regina* and *Lucilia illustris* (Meigen) in mixed forests in Maine (United States), and Goddard et al. (2020) documented *L. coeruleiviridis* moving 2 m eastward before radial dispersion in Mississippi (United States). Closer to our study region, observations from a human cadaver in the semi-arid Nearctic region of Coahuila, Mexico, reported a north to northwest orientation for a mixed calliphorid assemblage (comprising *L. sericata*, *Calliphora coloradensis* Hough, *P. regina*, *Co. macellaria*, and *Cochliomyia minima* Shannon) (Vergara-Pineda et al. 2012), contrasting with our Neotropical volcanic substrate findings. Out of the Americas, only Adetimehin et al. (2024) provides a comparative data from South African Mediterranean shrubland, where *Ch. albiceps* and *Ch. chloropyga* moved 1.5 m southeast following decomposition fluid gradients. This evidence concentrated in temperate ecosystems shows the necessity of more studies in other ecological zones such as tropical dry forests, cloud forests, and xeric shrublands, where higher year-round temperatures, extreme seasonal fluctuations in humidity, and intense solar radiation create markedly different decomposition dynamics than temperate regions. Furthermore, a crucial variable systematically omitted in these previous comparative studies is the initial physical position of the carcass. Given the species-specific behaviors (Ferreira et al. 2026) and negative phototaxis (Mactaggart et al. 2025) discussed earlier. Consequently, it remains unknown whether the reported directional vectors were purely driven by macro-environmental factors or were heavily biased by the carcass’s initial placement, reinforcing the need for controlled carcass orientation in future field experiments.

Our quantified dispersal metrics for *L. sericata* align with Greenberg’s (1990) laboratory classification of this species as an extensive disperser capable of 3 to 8 m movement, though our field observations under volcanic substrate conditions revealed more conservative dispersal distances for *L. sericata*. The bimodal distribution in our study showing primary peaks at 90 cm with isolated individuals of *P. casei* and *Ch. megacephala* reaching 250 cm suggests zone-specific dispersal influenced by substrate heterogeneity ranging from bulk density. This volcanic soil variation could create preferential pathways absent in homogeneous forest soils characteristic of Nearctic studies, while our observed interspecific directional convergence with *L. sericata* and *Ch. rufifacies* orienting northwest indicates local environmental factors override species behavioral movement. Standard forensic protocols recommending 2 to 10 m radial searches (Byrd and Tomberlin 2020) remain the baseline, our findings offer an opportunity to optimize research efficiency in Neotropical volcanic substrates. Given that substrate density gradients tightly concentrate larval dispersal compared to the homogeneous soils of temperate regions, investigators can prioritize highly sampling within an initial 2.5 m radius to early evidence recovery before expanding the search area.

### Temporal Plasticity and Adaptive Responses to Environmental Pressure

Temporal variation revealed behavioral plasticity with directional intensification during dry season versus dispersed patterns in wet season, contrasting with stability reported in laboratory conditions (Gomes et al. 2006). Active predation by and of the genus *Solenopsis* during dry season altered spatial larval movement, forcing in situ aggregation with over 500 pupae concentrated around specific carcass regions such as the throat. This behavioral modification under biotic pressure aligns with laboratory findings by Cammack et al. (2010), who demonstrated that *L. sericata* larvae alter their spatial distribution, specifically by puparating closer together, when exposed to the parasitoid wasp *Nasonia vitripennis* (Walker). Furthermore, these grouped patterns challenge the paradigm of purely individualistic movement. Recent evidence in Sarcophagidae suggests that such coordinated movements may represent true “dispersal aggregations” potentially mediated by chemical cues or pheromones as an adaptive life-history strategy (Ferreira et al. 2026), a dynamic that likely applies to the high-density formations we observed under severe environmental stress. However, our observed field scale, limited to 30 cm, suggests a severe spatial restriction. Secondary species *P. casei* and *N. littoralis* appeared exclusively during the wet season, suggesting a temporal preference for colonization.

### Forensic Implications and Optimization of Evidence Recovery in Neotropical Ecosystems

Convergent evidence from multiple studies showed that directionality transcends differences in carcass type with weights ranging from 3 to 725 kg (eg Lashley et al. 2018, Goddard et al. 2020). This independence extends to environment, manifesting from Mediterranean vegetation (Adetimehin et al. 2024), forests (Maisonhaute and Forbes 2021), grasslands (Tessmer and Meek 1996), and Neotropical volcanic substrates (the present study). In contrast, temporal variables emerge as critical determinants of directional behavior specifically during active larval migration. Morning hours consistently between 8 and 10 h documented by Goddard et al. (2020), Lewis and Benbow (2011), and our study represent specific ecological windows where active dispersal events peak. While standard entomological protocols establish that the recovery of static pupae or empty puparia from the soil is entirely independent of the time of day (Amendt et al. 2007), recognizing these morning peaks provides a specific advantage for intercepting actively dispersing larvae in early-stage investigations (Bambaradeniya et al. 2023). Although general forensic guidelines inherently favor daytime scene processing for visibility and to avoid artificial light artifacts, our findings highlight the early morning as the prime window for observing *in vivo* directional movement. This supports a nuanced operational understanding: while extensive radial soil sampling remains the indispensable standard for recovering static evidence regardless of the hour, recognizing active directional movement during clear mornings can serve as a valuable complementary tool for live specimen recovery if the investigation coincides with these temporal windows.

Based on our findings and the general nature of current standard protocols (eg Amendt et al. 2010, Byrd and Tomberlin 2020), these predictable spatiotemporal behaviors suggest potential opportunities for adapting forensic search protocols in similar ecosystems. Modern ecological interpretations increasingly highlight that dispersal behavior is not universal, but rather characterized by highly divergent, species-specific life-history strategies (Ferreira et al. 2026). Therefore, while an exhaustive radial search within a 3-m radius remains the indispensable standard under any condition (Amendt et al. 2007), investigators can leverage these patterns situationally. If active live dispersal is observed, which our data indicate peaks during clear wet season mornings, searches can be directionally extended toward the northwest (up to 5 m where terrain permits). Conversely, when dry season predatory pressure is evident, sampling efforts should be concentrated heavily in situ beneath the remains. This approach could help to balance thoroughness with operational efficiency, potentially maximizing recovery probability while minimizing investigation time in comparable Neotropical volcanic environments. However, validation across diverse sites and conditions would be necessary before widespread implementation.

The patterns documented in this study provide initial foundations for expanding research to enhance our understanding of directional behavior in necrophagous dipterans. Particularly promising are experimental studies comparing pupal weights to distinguish larval waves. Identifying these distinct cohorts is critical to avoid severe underestimations of the minPMI, as atypical dynamics like delayed oviposition or late-stage recolonization can easily be mistaken for primary colonization, especially when extensive dispersal obscures older pupal evidence (Owings et al. 2022). Furthermore, controlled carcass orientation experiments, and systematic light intensity measurements to explore phototaxis are recommended (Mactaggart et al. 2025). Additionally, detailed soil moisture monitoring could clarify how substrate saturation impacts survival and dispersal limits; saturated soils induce hypoxia (Bambaradeniya et al. 2024**)**, which significantly delays pupation, reduces adult biomass, and alters minPMI calculations (Kökdener and Yurtgan 2022, Bambaradeniya et al. 2024). These investigations, alongside the temporal tracking of linear behavior, characterization of physiological stimuli, and continued development of regional taxonomic databases, would contribute to strengthening Mexican forensic entomology. Overall, this study establishes quantitative post-feeding dispersal patterns for *L. sericata* under Neotropical volcanic conditions, demonstrating consistent northwest directionality (320°), ­spatially limited dispersal (95% of captured individuals within 2.9 m), and temporal plasticity with directional intensification during the dry season. These results provide empirical foundations for optimizing evidence recovery while identifying specific mechanistic research needs under comparable conditions.
